# Supplementary material for: The Role of rs713041 Glutathione Peroxidase 4 (GPX4) Single Nucleotide Polymorphism on Disease Susceptibility in Humans: A Systematic Review and Meta-Analysis
Source: Int J Mol Sci. 2022 Dec 12;23(24):15762. doi: 10.3390/ijms232415762 (PMC9778852; doi:10.3390/ijms232415762)
Supplement: Supplementary file 1 [file ijms-23-15762-s001.zip › Supplementary Figure S3 - Forest Plot Preeclampsia.pdf]

A

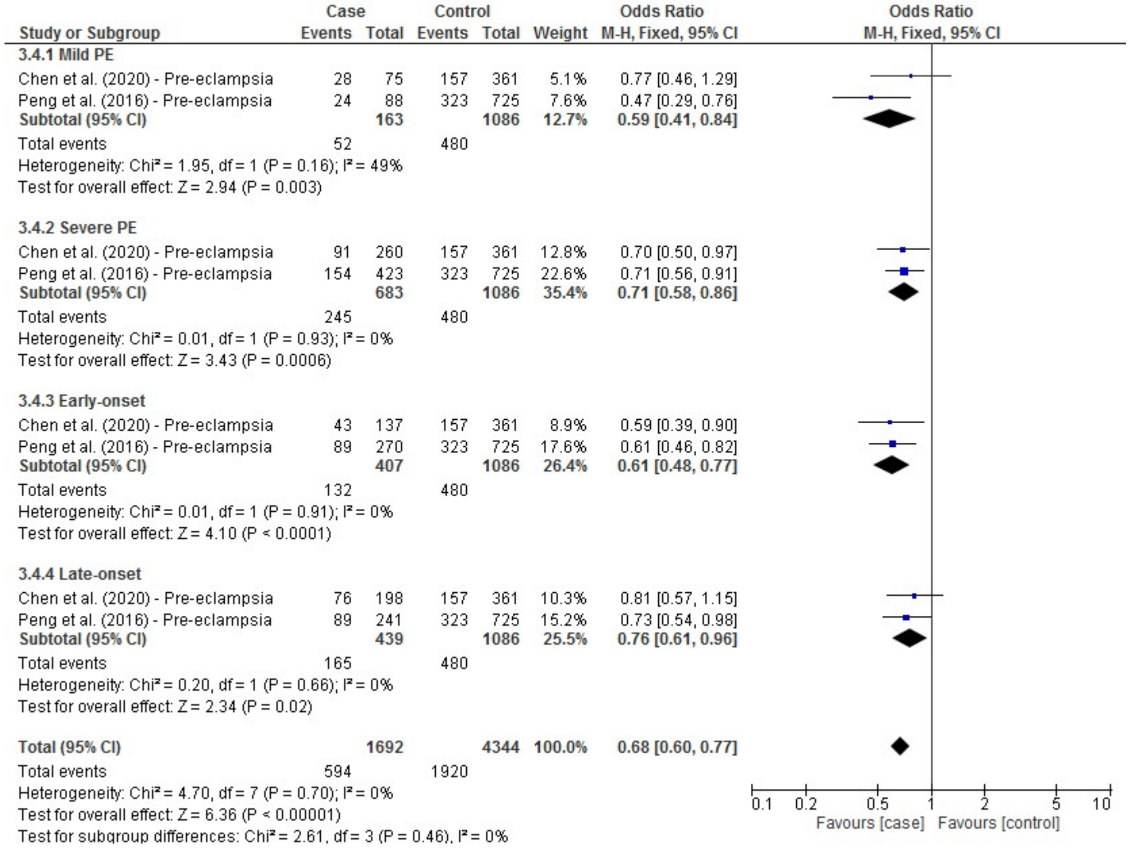
Study or Subgroup
Case Events Total
Control Events Total
Weight
Odds Ratio M-H, Fixed, 95% CI
Odds Ratio M-H, Fixed, 95% CI
3.4.1 Mild PE
Chen et al. (2020) - Pre-eclampsia 28 75 157 361 5.1% 0.77 [0.46, 1.29]
Peng et al. (2016) - Pre-eclampsia 24 88 323 725 7.6% 0.47 [0.29, 0.76]
Subtotal (95% CI) 163 1086 12.7% 0.59 [0.41, 0.84]
Total events 52 480
Heterogeneity: Chi² = 1.95, df = 1 (P = 0.16); I² = 49%
Test for overall effect: Z = 2.94 (P = 0.003)
3.4.2 Severe PE
Chen et al. (2020) - Pre-eclampsia 91 260 157 361 12.8% 0.70 [0.50, 0.97]
Peng et al. (2016) - Pre-eclampsia 154 423 323 725 22.6% 0.71 [0.56, 0.91]
Subtotal (95% CI) 683 1086 35.4% 0.71 [0.58, 0.86]
Total events 245 480
Heterogeneity: Chi² = 0.01, df = 1 (P = 0.93); I² = 0%
Test for overall effect: Z = 3.43 (P = 0.0006)
3.4.3 Early-onset
Chen et al. (2020) - Pre-eclampsia 43 137 157 361 8.9% 0.59 [0.39, 0.90]
Peng et al. (2016) - Pre-eclampsia 89 270 323 725 17.6% 0.61 [0.46, 0.82]
Subtotal (95% CI) 407 1086 26.4% 0.61 [0.48, 0.77]
Total events 132 480
Heterogeneity: Chi² = 0.01, df = 1 (P = 0.91); I² = 0%
Test for overall effect: Z = 4.10 (P < 0.0001)
3.4.4 Late-onset
Chen et al. (2020) - Pre-eclampsia 76 198 157 361 10.3% 0.81 [0.57, 1.15]
Peng et al. (2016) - Pre-eclampsia 89 241 323 725 15.2% 0.73 [0.54, 0.98]
Subtotal (95% CI) 439 1086 25.5% 0.76 [0.61, 0.96]
Total events 165 480
Heterogeneity: Chi² = 0.20, df = 1 (P = 0.66); I² = 0%
Test for overall effect: Z = 2.34 (P = 0.02)
Total (95% CI) 1692 4344 100.0% 0.68 [0.60, 0.77]
Total events 594 1920
Heterogeneity: Chi² = 4.70, df = 7 (P = 0.70); I² = 0%
Test for overall effect: Z = 6.36 (P < 0.00001)
Test for subgroup differences: Chi² = 2.61, df = 3 (P = 0.46), I² = 0%
0.1 0.2 0.5 1 2 5 10
Favours [case] Favours [control]

B

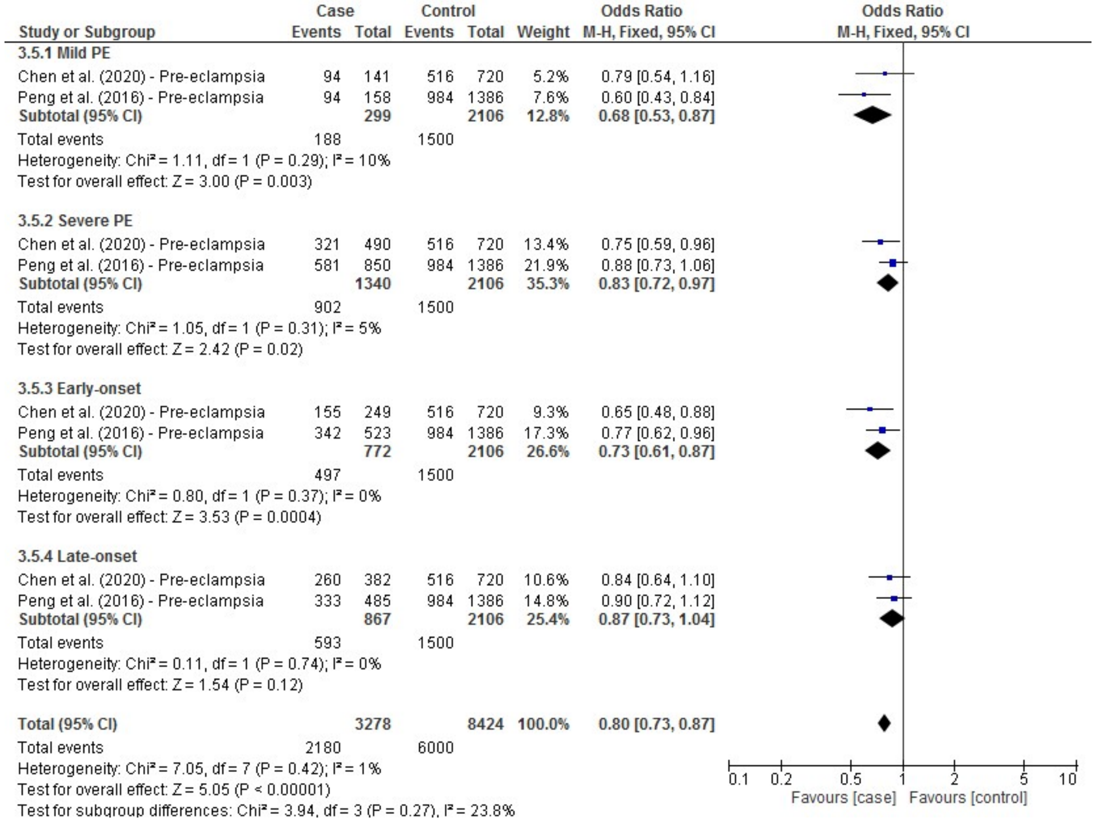
Study or Subgroup
Case Events Total
Control Events Total
Weight
Odds Ratio M-H, Fixed, 95% CI
Odds Ratio M-H, Fixed, 95% CI
3.5.1 Mild PE
Chen et al. (2020) - Pre-eclampsia 94 141 516 720 5.2% 0.79 [0.54, 1.16]
Peng et al. (2016) - Pre-eclampsia 94 158 984 1386 7.6% 0.60 [0.43, 0.84]
Subtotal (95% CI) 299 2106 12.8% 0.68 [0.53, 0.87]
Total events 188 1500
Heterogeneity: Chi² = 1.11, df = 1 (P = 0.29); I² = 10%
Test for overall effect: Z = 3.00 (P = 0.003)
3.5.2 Severe PE
Chen et al. (2020) - Pre-eclampsia 321 490 516 720 13.4% 0.75 [0.59, 0.96]
Peng et al. (2016) - Pre-eclampsia 581 850 984 1386 21.9% 0.88 [0.73, 1.06]
Subtotal (95% CI) 1340 2106 35.3% 0.83 [0.72, 0.97]
Total events 902 1500
Heterogeneity: Chi² = 1.05, df = 1 (P = 0.31); I² = 5%
Test for overall effect: Z = 2.42 (P = 0.02)
3.5.3 Early-onset
Chen et al. (2020) - Pre-eclampsia 155 249 516 720 9.3% 0.65 [0.48, 0.88]
Peng et al. (2016) - Pre-eclampsia 342 523 984 1386 17.3% 0.77 [0.62, 0.96]
Subtotal (95% CI) 772 2106 26.6% 0.73 [0.61, 0.87]
Total events 497 1500
Heterogeneity: Chi² = 0.80, df = 1 (P = 0.37); I² = 0%
Test for overall effect: Z = 3.53 (P = 0.0004)
3.5.4 Late-onset
Chen et al. (2020) - Pre-eclampsia 260 382 516 720 10.6% 0.84 [0.64, 1.10]
Peng et al. (2016) - Pre-eclampsia 333 485 984 1386 14.8% 0.90 [0.72, 1.12]
Subtotal (95% CI) 867 2106 25.4% 0.87 [0.73, 1.04]
Total events 593 1500
Heterogeneity: Chi² = 0.11, df = 1 (P = 0.74); I² = 0%
Test for overall effect: Z = 1.54 (P = 0.12)
Total (95% CI) 3278 8424 100.0% 0.80 [0.73, 0.87]
Total events 2180 6000
Heterogeneity: Chi² = 7.05, df = 7 (P = 0.42); I² = 1%
Test for overall effect: Z = 5.05 (P < 0.00001)
Test for subgroup differences: Chi² = 3.94, df = 3 (P = 0.27), I² = 23.8%
0.1 0.2 0.5 1 2 5 10
Favours [case] Favours [control]

C

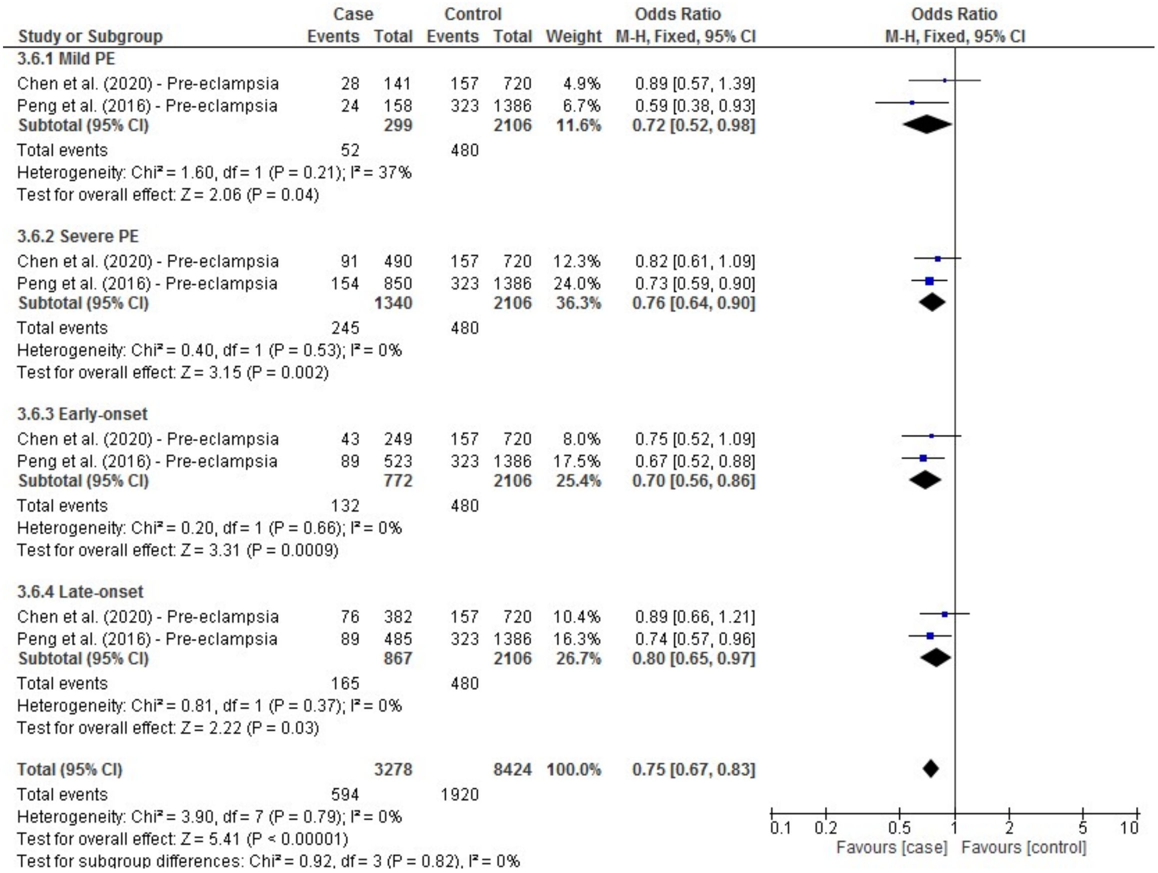

Study or Subgroup	Case Events	Case Total	Control Events	Control Total	Weight	Odds Ratio M-H, Fixed, 95% CI
**3.6.1 Mild PE**						
Chen et al. (2020) - Pre-eclampsia	28	141	157	720	4.9%	0.89 [0.57, 1.39]
Peng et al. (2016) - Pre-eclampsia	24	158	323	1386	6.7%	0.59 [0.38, 0.93]
**Subtotal (95% CI)**		**299**		**2106**	**11.6%**	**0.72 [0.52, 0.98]**
Total events	52		480			
Heterogeneity: Chi² = 1.60, df = 1 (P = 0.21); I² = 37%						
Test for overall effect: Z = 2.06 (P = 0.04)						
**3.6.2 Severe PE**						
Chen et al. (2020) - Pre-eclampsia	91	490	157	720	12.3%	0.82 [0.61, 1.09]
Peng et al. (2016) - Pre-eclampsia	154	850	323	1386	24.0%	0.73 [0.59, 0.90]
**Subtotal (95% CI)**		**1340**		**2106**	**36.3%**	**0.76 [0.64, 0.90]**
Total events	245		480			
Heterogeneity: Chi² = 0.40, df = 1 (P = 0.53); I² = 0%						
Test for overall effect: Z = 3.15 (P = 0.002)						
**3.6.3 Early-onset**						
Chen et al. (2020) - Pre-eclampsia	43	249	157	720	8.0%	0.75 [0.52, 1.09]
Peng et al. (2016) - Pre-eclampsia	89	523	323	1386	17.5%	0.67 [0.52, 0.88]
**Subtotal (95% CI)**		**772**		**2106**	**25.4%**	**0.70 [0.56, 0.86]**
Total events	132		480			
Heterogeneity: Chi² = 0.20, df = 1 (P = 0.66); I² = 0%						
Test for overall effect: Z = 3.31 (P = 0.0009)						
**3.6.4 Late-onset**						
Chen et al. (2020) - Pre-eclampsia	76	382	157	720	10.4%	0.89 [0.66, 1.21]
Peng et al. (2016) - Pre-eclampsia	89	485	323	1386	16.3%	0.74 [0.57, 0.96]
**Subtotal (95% CI)**		**867**		**2106**	**26.7%**	**0.80 [0.65, 0.97]**
Total events	165		480			
Heterogeneity: Chi² = 0.81, df = 1 (P = 0.37); I² = 0%						
Test for overall effect: Z = 2.22 (P = 0.03)						
**Total (95% CI)**		**3278**		**8424**	**100.0%**	**0.75 [0.67, 0.83]**
Total events	594		1920			
Heterogeneity: Chi² = 3.90, df = 7 (P = 0.79); I² = 0%						
Test for overall effect: Z = 5.41 (P < 0.00001)						
Test for subgroup differences: Chi² = 0.92, df = 3 (P = 0.82), I² = 0%						

Supplementary Figure S3: Forest plots of odds ratios with 95% confidence intervals of the association between *GPX4* (rs713041) SNP and risk of mild, severe, early-onset and late-onset PE. (**A**) - Additive model (TT vs CC) [38,39], (**B**) - Dominant model (CT+TT vs CC) [38,39] and (**C**) - Recessive model (TT vs CC+CT) [38,39].
