# Supplementary material for: The Role of rs713041 Glutathione Peroxidase 4 (GPX4) Single Nucleotide Polymorphism on Disease Susceptibility in Humans: A Systematic Review and Meta-Analysis
Source: Int J Mol Sci. 2022 Dec 12;23(24):15762. doi: 10.3390/ijms232415762 (PMC9778852; doi:10.3390/ijms232415762)
Supplement: Supplementary file 1 [file ijms-23-15762-s001.zip › Supplementary Figure S4 - Funnel plot-reproductive.pdf]

A

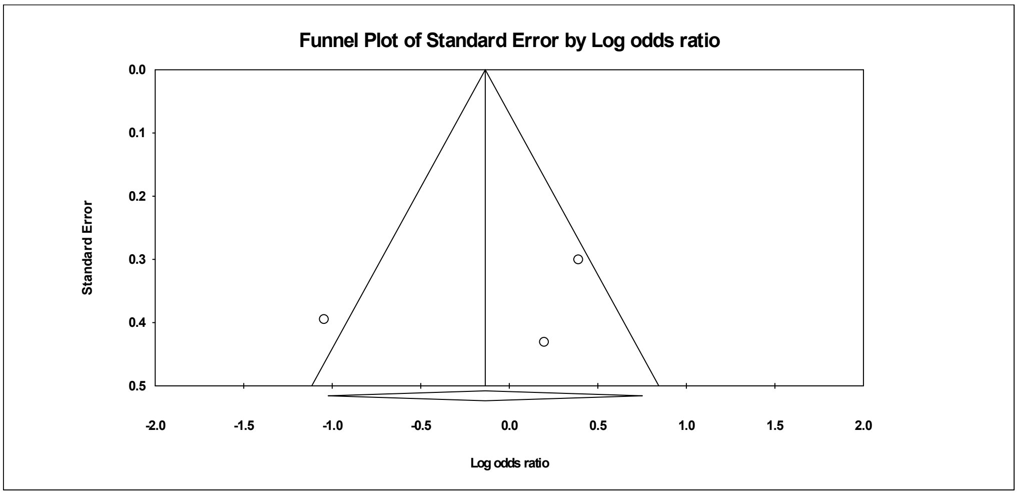

B

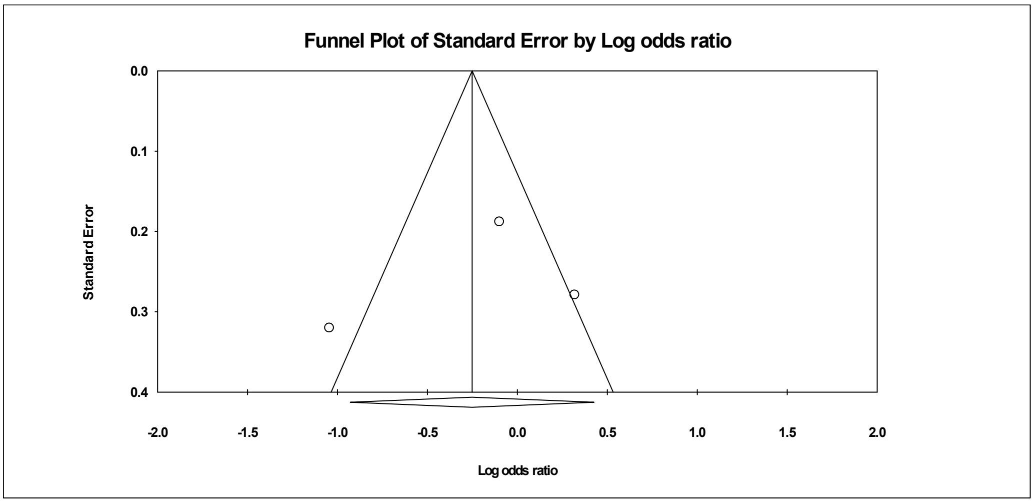

C

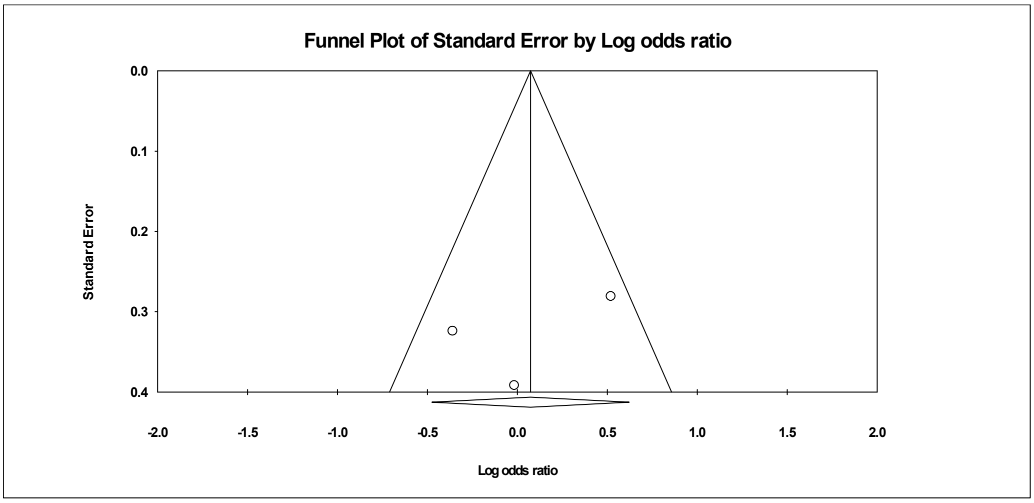

Supplementary Figure S4: Funnel plots of the association between *GPX4* (rs713041) SNP and risk of reproduction-related diseases. (**A**) - Additive model (TT vs CC) [22,42,43], (**B**) - Dominant model (CT+TT vs CC) [22,42,43] and (**C**) - Recessive model (TT vs CC+CT) [22,42,43].
