# Supplementary material for: The Role of rs713041 Glutathione Peroxidase 4 (GPX4) Single Nucleotide Polymorphism on Disease Susceptibility in Humans: A Systematic Review and Meta-Analysis
Source: Int J Mol Sci. 2022 Dec 12;23(24):15762. doi: 10.3390/ijms232415762 (PMC9778852; doi:10.3390/ijms232415762)
Supplement: Supplementary file 1 [file ijms-23-15762-s001.zip › Supplementary Figure S5 - Funnel plot-selenium.pdf]

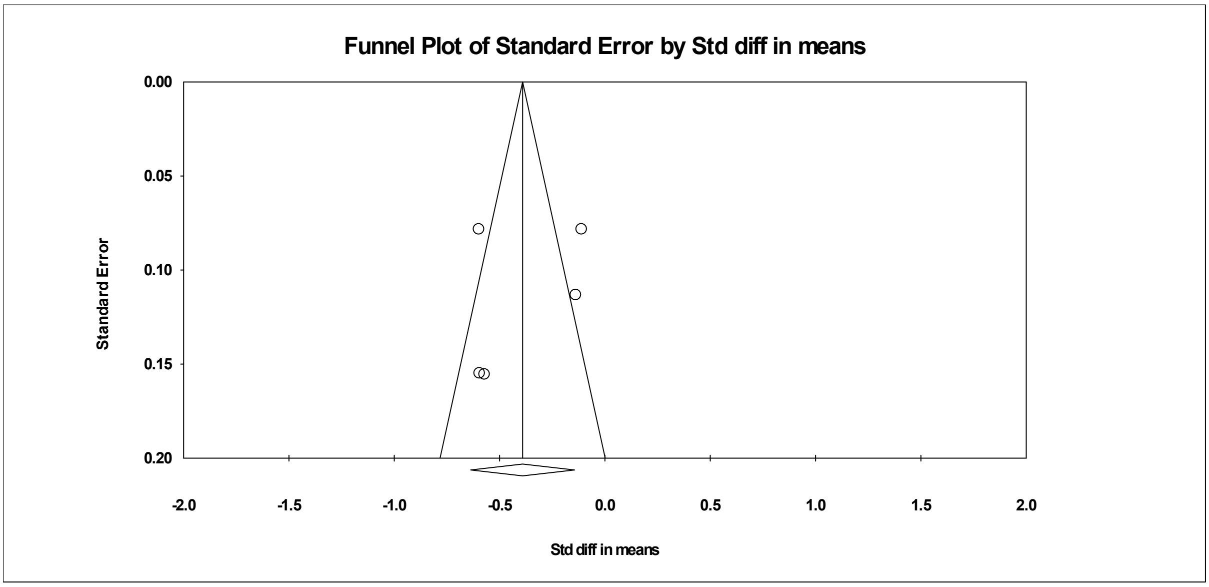

Supplementary Figure S5: Funnel plot standardised mean difference (SMD) of selenium levels between cases and controls [17,19, 20, 37].
