# Supplementary material for: The Role of rs713041 Glutathione Peroxidase 4 (GPX4) Single Nucleotide Polymorphism on Disease Susceptibility in Humans: A Systematic Review and Meta-Analysis
Source: Int J Mol Sci. 2022 Dec 12;23(24):15762. doi: 10.3390/ijms232415762 (PMC9778852; doi:10.3390/ijms232415762)
Supplement: Supplementary file 1 [file ijms-23-15762-s001.zip › Supplementary Table S2 - String Table.pdf]

Table S2: Search string used for retrieving studies in selected databases.

Database	Search String
CINAHL	"GPX4" OR "Glutathione peroxidase 4" OR "Phospholipid glutathione peroxidase" OR "PH-GSH" OR "GPX-4" AND "Polymorphism*" OR "rs713041" OR "GPx4 TC 718" OR "SNP" OR "genetic" OR "variant" OR "SNP" OR "single nucleotide polymorphism" OR "mutation – Boolean/Phrase
Cochrane	"GPX4" OR "Glutathione peroxidase 4" OR "Phospholipid glutathione peroxidase" OR "PH-GSH" OR "GPX-4" in Title Abstract Keyword AND "Polymorphism*" OR "rs713041" OR "GPx4 TC 718" OR "SNP" OR "genetic" OR "variant" OR "SNP" OR "single nucleotide polymorphism" OR "mutation" in Title Abstract Keyword
Medline	"GPX4" OR "Glutathione peroxidase 4" OR "Phospholipid glutathione peroxidase" OR "PH-GSH" OR "GPX-4" AND "Polymorphism*" OR "rs713041" OR "GPx4 TC 718" OR "SNP" OR "genetic" OR "variant" OR "SNP" OR "single nucleotide polymorphism" OR "mutation – Boolean/Phrase
SCOPUS	(TITLE-ABS-KEY) "GPX4" OR "Glutathione peroxidase 4" OR "Phospholipid glutathione peroxidase" OR "PH-GSH") AND (TITLE-ABS-KEY) "Polymorphism*" OR "rs713041" OR "GPx4 T/C 718" OR "SNP" OR "genetic" OR "variant" OR "SNP" OR "single nucleotide polymorphism" OR "mutation"
PUBMED	"GPX4" OR "Glutathione peroxidase 4" OR "Phospholipid glutathione peroxidase" OR "PH-GSH" OR "GPX-4") AND "Polymorphism*" OR "rs713041" OR "GPx4 T/C 718" OR "SNP" OR "genetic" OR "variant" OR "SNP" OR "single nucleotide polymorphism" OR "mutation")
Web of Science	"GPX4" OR "Glutathione peroxidase 4" OR "Phospholipid glutathione peroxidase" OR "PH-GSH" OR "GPX-4" (Topic) AND "Polymorphism*" OR "rs713041" OR "GPx4 TC 718" OR "SNP" OR "genetic" OR "variant" OR "SNP" OR "single nucleotide polymorphism" OR "mutation" (Topic)
