# Supplementary material for: The Role of rs713041 Glutathione Peroxidase 4 (GPX4) Single Nucleotide Polymorphism on Disease Susceptibility in Humans: A Systematic Review and Meta-Analysis
Source: Int J Mol Sci. 2022 Dec 12;23(24):15762. doi: 10.3390/ijms232415762 (PMC9778852; doi:10.3390/ijms232415762)
Supplement: Supplementary file 1 [file ijms-23-15762-s001.zip › Supplementary Table S3 - Newcastle-Ottawa Quality Assessment Scale.pdf]

Table S3: Quality of individual studies included in the meta–analysis according to Newcastle-Ottawa scale for case-control studies

**Author (Year)**	**Disease**	**Selection**				**Comparability**		**Exposure**			**Total score**
		Is the case definition adequate?	Representativeness of the cases	Selection of controls	Definition of controls	Most important factor - **Genotype**	Additional factor	Ascertainment of exposure	Same method of ascertainment for cases and controls	Non-response rate	
Peters (2008) [35]	Advanced distal colorectal adenoma	*	*		*	*	*	*	*	*	8 stars
Méplan (2010) [34]	Colorectal cancer	*	*		*	*		*	*	*	7 stars
Steinbrecher (2010) [36]	Prostate cancer	*	*		*	*	*	*	*	*	8 stars
Du (2012) [45]	Kashin-Beck disease	*	*		*	*	*	*	*	*	8 stars
Karunasinghe (2012) [20]	Prostate cancer	*	*	*	*	*	*	*	*	*	9 stars
Jaworska (2013) [19]	Laryngeal cancer	*	*	*	*	*	*	*	*	*	9 stars
	Lung cancer	*	*	*	*	*	*	*	*	*	9 stars
Méplan (2013) [16]	Breast cancer	*	*		*	*	*	*	*	*	8 stars
Khadzhieva (2014) [41]	Recurrent miscarriage	*	*		*	*		*	*	*	7 stars
Jablonska (2015) [17]	Breast cancer	*	*		*	*	*	*	*	*	8 Stars
Peng (2016) [38]	Pre-eclampsia	*	*		*	*		*	*	*	7 stars
Xiao (2017) [23]	Thyroid diseases	*	*		*	*		*	*	*	7 stars
da Rocha (2018) [24]	Alzheimer disease	*	*		*	*		*	*	*	7 stars
Wigner (2018) [25]	Depression	*	*		*	*		*	*	*	7 stars
Chen (2020) [37]	Pre-eclampsia	*	*		*	*		*	*	*	7 stars
Corredor (2020) [39]	Hypertension	*	*		*	*		*	*	*	7 stars
Huang (2020) [22]	Endometriosis	*	*		*	*		*	*	*	7 stars
Mashkina (2020) [42]	Pregnancy loss	*	*		*	*		*	*	*	7 stars
Gusti (2021) [44]	Type 2 diabetes mellitus	*	*		*	*	*	*	*	*	8 stars

Synowiec (2021) [40]	Ischemic stroke	*	*		*	*		*	*	*	7 stars
Ściskalska (2022) [43]	Acute pancreatitis	*	*		*	*	*	*	*	*	8 stars
Wigner (2022) [26]	Multiple sclerosis	*	*		*	*		*	*	*	7 stars

Studies that scored >8 were classified of high quality, 4-7 of moderate quality and <3 of poor quality
